# Can household clean energy transition reduce medical expenditures? Evidence from China

**DOI:** 10.3389/fpubh.2025.1524444

**Published:** 2025-08-15

**Authors:** Tao Li, Dan Xiao Yang

**Affiliations:** College of Economics, Qingdao University, Qingdao, China

**Keywords:** household clean energy transition, medical expenditures, clean energy policy, staggered DID, CFPS, China

## Abstract

Indoor air pollution is a significant issue in developing nations, posing serious health risks and contributing to various diseases. Despite its importance, the relationship between household clean energy transition and its effects on health outcomes and medical expenses has received limited scholarly attention. This study addresses this gap by utilizing the Air Pollution Prevention and Control Action Plan (APCP), implemented by the State Council of China in 2013, as a policy intervention. Using data from the China Family Panel Studies (CFPS) spanning from 2014 to 2020, a staggered Difference-in-Differences (DID) model was developed to assess the impact of the transition to clean energy on medical expenses. The findings indicate that household clean energy transition can significantly reduce residents’ medical expenditures. Heterogeneity analyses indicate that rural populations, individuals with lower educational attainment, homeowners, and families consisting of three to five members experience a more significant reduction in medical costs associated with household clean energy transition. Mechanism analysis reveals that the reduction in medical costs is attributable to the improvements in health outcomes and increases in income resulting from the clean energy transition. This study offers a significant academic foundation and support for developing countries in formulating clean energy and health policies.

## 1 Introduction

Indoor air pollution remains a pressing issue in numerous regions across China. Coal, wood, and crop residues have long served as significant energy sources for heating and cooking. When burned, these fuels release a substantial amount of pollutants. The harmful substances generated can lead to non - communicable diseases such as stroke, ischemic heart disease, chronic obstructive pulmonary disease (COPD), and lung cancer (1). According to a report from the International Energy Agency (IEA), approximately 1.2 million people in China die prematurely due to indoor air pollution, a figure significantly higher than the number of deaths caused by outdoor air pollution (2). Therefore, transitioning to clean household energy is crucial for mitigating the harmful effects of indoor air pollution. In line with the 2030 Agenda for Sustainable Development and the pursuit of health equity, the United Nations has advocated for replacing traditional polluting fuels with cleaner energy alternatives. The Chinese government has taken proactive actions. On September 10, 2013, it formulated the Air Pollution Prevention and Control Action Plan, promoting measures such as “coal-to-gas” and “coal-to-electricity” conversions and increasing natural gas use. These efforts have significantly improved the energy consumption structure of Chinese households, reduced pollutant gas emissions, and brought about enormous health benefits (3).

Indoor air pollution poses significant risks to public health and contributes to rising medical expenditures. Currently, approximately 2 billion individuals worldwide are experiencing financial strain due to medical costs, which impedes timely access to medical services and adversely affects health outcomes, human capital development, and, ultimately, household income generation (4). Insufficient income often fails to cover the high costs of medical care, thereby trapping individuals in a “vicious cycle of poverty” (5). In light of this situation, the primary objective of this paper is to examine the potential effects of transition to clean household energy on medical costs, as well as to explore the underlying mechanisms involved.

Current research on energy transition within the household sector predominantly focuses on factors such as labor force participation and health outcomes. For example, Khandker et al. utilized data from the Indian Human Development Survey (IHDS) to demonstrate that rural electrification leads to an increase in employment hours, resulting in a 38.6% rise in per capita household income (6). Zhang et al. employed a continuous-space difference-in-differences (DID) model to examine the impact of clean heating policies on employment. The findings reveal that the transition to clean heating significantly increases total employment in local cities and their neighboring areas. This effect is driven by the following pathways: the implementation of clean heating policies contributes to improved air quality, stimulates investment in heating systems, and effectively promotes employment growth through technological innovation (7). Environmental pollution increases the likelihood of physical discomfort and the risk of chronic diseases, thereby posing a threat to people’s physical health (8). Barron, M. et al. further contributed to this discourse by analyzing the effects of household electrification on indoor air quality, concluding that the transition from coal to electricity significantly reduces indoor PM2.5 levels and subsequently decreases the prevalence of ARI among school-age children (9). Liao, L. et al. utilized data from the China Family Panel Studies (CFPS) to investigate the impact of clean heating policies on individual health. The findings revealed that the implementation of clean heating policies increased the probability of improved air quality by 17.4% and enhanced public awareness of environmental protection, significantly contributing to better health outcomes (10).

However, there is a relative scarcity of studies examining the implications of household clean energy transitions on medical expenditures. Lin, B. and K. Wei conducted an analysis of the impact of household use of solid fuels on medical expenditures, utilizing the OLS regression estimation. Their findings indicate that households relying on solid cooking fuels experience a significantly higher incidence of respiratory diseases among their members due to chronic exposure to more polluted environments and tend to incur a heavier burden of medical costs compared to households that utilize cleaner and more modern cooking methods. However, the authors did not explore the policy implications of household energy transitions on medical costs, and the methodological approach employed raises certain endogeneity concerns (11). In light of these gaps, this paper presents two key innovations. First, it investigates the effects of household clean energy transitions on medical expenditures through the lens of clean energy policy and elucidates the mechanisms underlying this impact, thereby enriching the existing literature on household energy transitions and medical expenses. Second, this study examines the policy implications of household clean energy transitions on medical costs while addressing specific endogeneity issues by employing a staggered DID methodology.

This study provides a comprehensive analysis of the impact of households’ transition to clean energy on medical expenditures, utilizing data from the CFPS conducted between 2014 and 2020. The findings reveal that household energy transition can reduce medical costs, with a noted decrease of 16.1% after controlling for fixed effects and variables. The results are robust, having passed various stability tests, including placebo test, parallel trend assessment, and the PSM-DID estimation. Furthermore, mechanism analysis reveals that the reduction in medical costs is mediated by the improvements in public health and increases in income. The study also examines regional, educational, homeownership, and household size heterogeneities to assess the differential impacts of the clean energy transition on medical costs. It concludes that the transition is particularly effective in lowering medical costs among rural populations, individuals with lower educational attainment, homeowners, and households consisting of three to five members.

The organization of the forthcoming sections of this study is outlined as follows: Section 2 provides the contextual background for the plan and the research hypotheses related to the underlying mechanisms involved. Section 3 outlines the research design. Section 4 discusses the regression results, and Section 5 concludes with the findings and their implications for policy.

## 2 An overview of the household clean energy transition program in China

### 2.1 Household clean energy transition program in China

Throughout the duration of the 12th Five-Year Plan, air pollution in China escalated to concerning levels, with regional atmospheric environmental challenges—especially those associated with PM10 and PM2.5—becoming increasingly evident. These challenges posed significant risks to public health and undermined social harmony and stability. According to the report titled China’s Environmental Protection Situation and Countermeasures, early 2013, widespread pollution affected nearly 2.7 million square kilometers of land—over one-quarter of the nation’s total area. This pollution affected 17 provinces and regions, encompassing more than 40 major cities and a population of approximately 600 million individuals. In response to this critical situation, the State Council of China introduced the APCP on September 10, 2013, aimed at improving air quality and promoting sustainable and healthy economic development.

In accordance with the policy, household energy transition will be facilitated through the implementation of clean energy measures targeting coal. These measures include the establishment of high-pollution no-burn zones, the promotion of electricity and natural gas as alternatives to coal, and the utilization of clean coal and coal-type fuels in rural areas of northern China, accompanied by the provision of appropriate subsidies. A statistical study conducted in 2013 by the Environmental Planning Institute in China revealed that the contribution of coal usage to specific environmental pollutants is estimated to be between 50 and 60%. Therefore, controlling the total amount of coal consumption is a crucial strategy for reducing air pollution. Concurrently, the APCP underscores the necessity of accelerating the restructuring of the energy framework and improving the availability of clean energy sources. For instance, increasing the supply of natural gas, coal-based natural gas, and coalbed methane can help reduce pollution emissions, while prioritizing the introduction of new natural gas sources to ensure residential use or to substitute for coal combustion.

**Figure 1:**
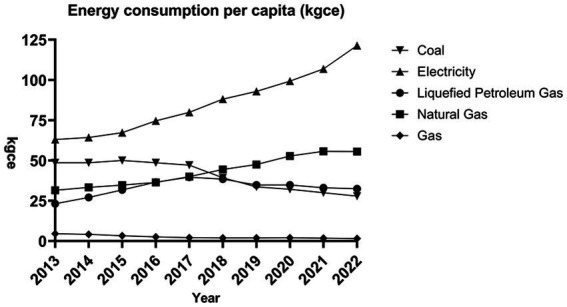
Per capita energy consumption structure chart.

The gradual implementation of the APCP has resulted in significant changes in the energy consumption patterns of Chinese households. This study employs per capita living energy consumption data from the China Energy Statistical Yearbook, covering the years from 2013 to 2023, to construct Figure 1. The data presented in Figure 1 highlight the increasing dominance of electric energy in residential consumption, while the use of coal and gas has shown a consistent decline over the years. Furthermore, natural gas is progressively replacing coal as the secondary energy source, in accordance with policy directives. Additionally, LPG, which functions as a transitional fuel, experienced a gradual increase in consumption until 2017, after which its usage began to decrease. These findings emphasize the significant impact of the APCP in promoting the transition toward cleaner energy sources for households.

### 2.2 Literature review

#### 2.2.1 Health effect

The “Action Plan for Air Pollution Prevention and Control” aims to enhance the accessibility of clean energy for Chinese households, which will effectively improve the energy consumption structure for household cooking and heating. As cleaner energy sources such as natural gas and electricity gradually replace solid fuels like wood and coal, indoor air quality will be enhanced, thereby generating significant health benefits. Li et al. conducted research based on the clean energy policy promulgated by the Chinese government in 2017 and found that the transition to clean energy can reduce emissions of PM2.5 and CO, significantly improving air quality (12). The improvement in air quality reduces the likelihood of health deterioration by 3.8%. Moreover, the transition from traditional fuels to natural gas has a more pronounced impact on health compared to the shift towards electricity (13).

In addition, data from the 2017 Medical Expenditure Panel Survey (MEPS) in the United States indicate a strong negative correlation between self-reported health status and medical expenses, meaning that individuals with poor health status incur medical expenses that are three to five times higher than those with good health status (14). Building on this, several scholars have discovered that compared to individuals with higher health literacy, those with lower health literacy have more frequent outpatient visits and higher hospitalization costs (15, 16). Based on the aforementioned research findings, we propose Hypothesis 1.

*H1: Household clean energy transition can enhance public health and subsequently lower medical expenditures.*

#### 2.2.2 Income effect

The impact of household clean energy transition on medical expenditures is also reflected in the fact that the transition can enhance people’s income, thereby reducing medical costs on the basis of increased income. The use of electricity and natural gas enables individuals to devote more time to social labor. For instance, Salmon, C. and J. Tanguy found that household electrification can reduce women’s time spent on household chores, thereby increasing their labor force participation (17). Similarly, scholars have discovered that the adoption of clean energy reduces the time spent on collecting solid fuels, leading to an increase of 3–5 h in weekly working hours and subsequently boosting household income (6, 18).

With an increased income, people are more likely to prioritize a balanced nutritional intake, improving their dietary quality (19), which is conducive to better health outcomes (20). Consequently, this reduces the frequency of hospital visits and lowers medical expenses. Additionally, higher income has a positive effect on the purchase of preventive healthcare (21), such as increasing the uptake of health insurance. By spending a relatively small amount, individuals can obtain substantial health protection, facilitating timely disease detection and early treatment. The reimbursement of medical expenses significantly reduces overall healthcare costs. In light of this analysis, we propose Hypothesis 2.

*H2: Household clean energy transition can increase individuals’ income levels and subsequently lower medical expenditures.*

**Figure 2:**
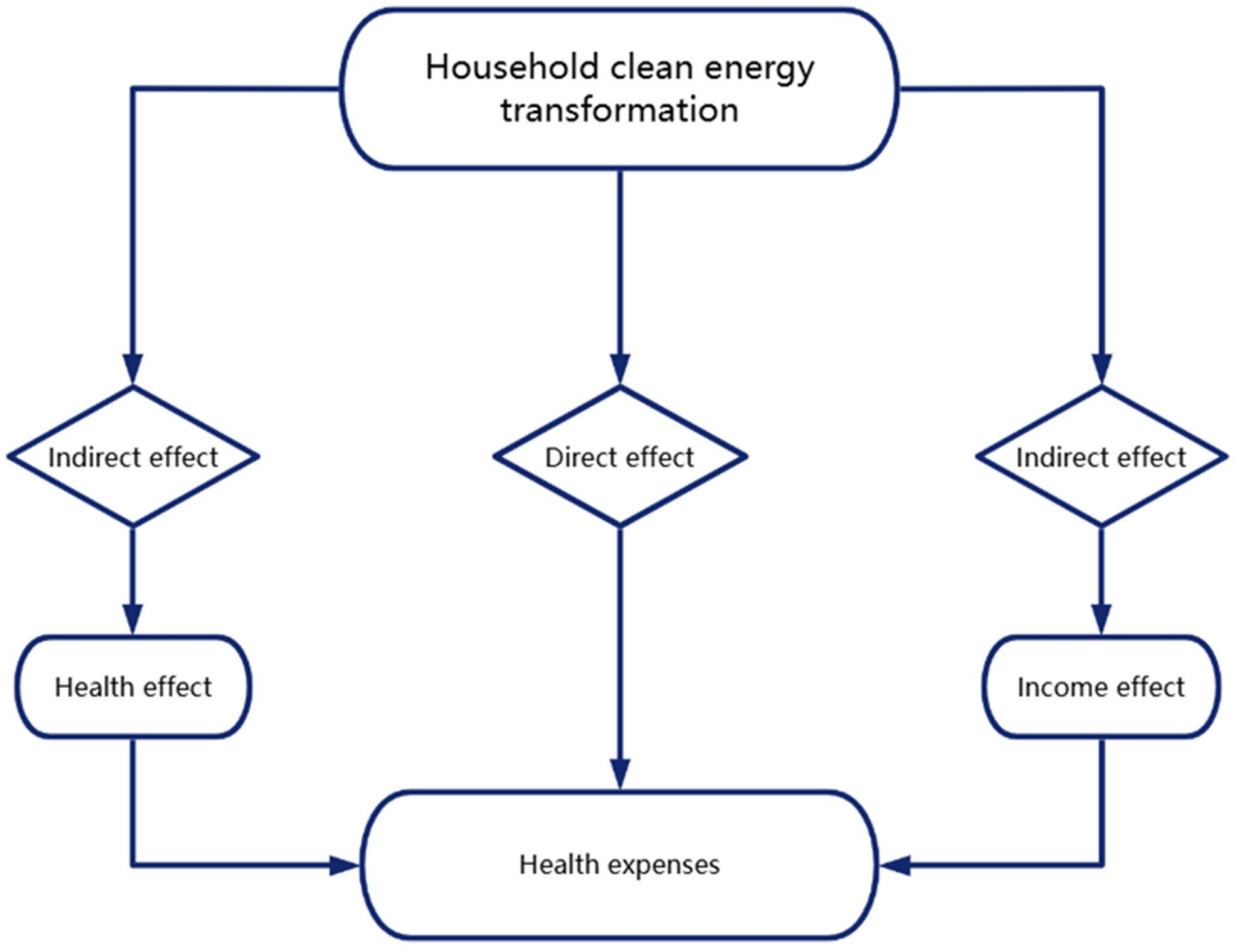
Mechanism framework diagram.

According to the preceding analysis, the impact of household clean energy transition on medical expenditures primarily occurs indirectly, mediated by health-related outcomes and changes in income. The mechanisms underlying this relationship are illustrated in Figure 2.

## 3 Research design

### 3.1 Data and variable selection

The data used in this study primarily originate from the CFPS, designed to capture the current status and dynamics of various aspects of Chinese society, including its economy, demographics, educational attainment, and health conditions. This is achieved by systematically collecting data at three levels: individual, household, and community. The dataset encompasses six distinct periods, specifically the years 2010, 2012, 2014, 2016, 2018, and 2020. Given the relevance of household clean energy transition indicators, this research focuses on data from four specific periods, namely from 2014 to 2020. The study involves the integration of individual and household databases through the use of unique identifiers for individuals and households, thereby facilitating the analysis of characteristics pertinent to the research. Subsequently, we identified individuals who were consistently tracked across the four survey periods from 2014 to 2020. Following a thorough process of data cleaning and matching, we ultimately obtained a dataset comprising 10,820 valid observations.

The independent variable in this study is medical expense, defined as the total household medical expenditures reported by the respondent divided by the total number of individuals in the household, resulting in a per capita medical cost. To reduce the impact of outliers, the resulting values underwent logarithmic transformation.

In this study, we build upon prior research findings and identify a total of nine control variables. These variables include an individual’s age and its squared term to account for the non-linear relationship associated with age, gender and urban–rural residence status to address potential gender and regional disparities that may influence medical expenditures, years of education to assess the impact of personal endowment on medical costs, and factors such as the presence of health insurance, self-reported health status, level of medical institutions, and the existence of chronic diseases. These variables are included to mitigate the influence of health insurance policies, individual health conditions, and medical utilization on medical expenses. This study also incorporates several control variables, including health insurance coverage, self-reported health status, the level of medical institutions accessed, and the existence of chronic diseases. The inclusion of these variables aims to mitigate the influence of health insurance policies, individual health conditions, and medical utilization on medical expenditures. These variables are defined in Table 1.

**Table 1:** Definition of main variables.

Variables	Variables	Definition
Dependent variable	fp511	Logarithm of medical expenses
Control variables	gender	Male = 1, female = 0
	age	Respondents reported age
	age2	Age squared term /100
	qa301	Urban = 1, rural = 0
	edu	Years of education
	qp605	Have basic health insurance = 1, otherwise = 0
	qp201	health level (1, lowest;5, highest)
	qp203	Level of medical institutions (1, lowest;5, highest)
	qp204	Any chronic diseases = 1, otherwise = 0

### 3.2 Descriptive statistics

In accordance with the clean energy indicators established by scholars (22), we assessed whether the households of respondents had successfully undergone a clean energy transition by analyzing their reported energy sources used for home cooking. Respondents were categorized into the treatment group if they utilized clean energy sources (such as electricity or natural gas) during the study period, while those who relied on non-clean energy sources (including coal, firewood, or kerosene) were assigned to the control group. The descriptive statistics pertaining to the sample are presented in Table 2. The treatment group exhibited significantly lower medical costs on average compared to the control group, suggesting that the household clean energy transition may contribute to a reduction in individual medical expenses. Furthermore, respondents in the treatment group were predominantly urban households and possessed a higher level of education relative to those in the control group. Additionally, when selecting medical institutions, individuals in the treatment group demonstrated a preference for more specialized medical facilities, and their overall health status was generally better than that of the control group.

**Table 2:** Descriptive statistics.

Variables	(1)	(2)	(3)	(4)	(5)	(6)
	Obs	Mean	Std. Dev.	Obs	Mean	Std. Dev.
	Treated group			Control group		
fp511	10,820	5.390	2.444	4,600	5.581	2.217
gender	10,820	0.454	0.498	4,600	0.457	0.498
age	10,820	31.27	6.644	4,600	31.63	6.675
age2	10,820	10.22	4.125	4,600	10.45	4.136
qa301	10,820	0.312	0.463	4,600	0.161	0.368
edu	10,820	10.91	4.090	4,600	9.551	4.296
qp605	10,820	0.898	0.302	4,600	0.907	0.291
qp201	10,820	3.383	1.045	4,600	3.361	1.076
qp203	10,820	3.138	1.637	4,600	2.884	1.553
qp204	10,820	0.0691	0.254	4,600	0.0717	0.258

### 3.3 Model setting

#### 3.3.1 Staggered DID model

This paper employs a staggered DID model to evaluate the effects of household clean energy transition on medical expenditures. We utilize the APCP, as a quasi-natural experiment to analyze variations in the average medical costs incurred by residents before and after the transition to clean energy in households. The specific model is detailed in Equation (1):


(1) $$Y_{\mathrm{it}}=\beta_{0}+\beta_{1}\text{Clean}\_\mathrm{us}e_{\mathrm{it}}+\beta_{2}\text{Contro}l_{\mathrm{it}}+\mu_{\rho }+\lambda_{t}+\varepsilon_{\mathrm{it}}$$

Y_it_ denotes individual i’s health expenditure in year t. Clean_use_it_ denotes the household clean energy use of individual i in year t. Clean_use_it_ = 1 if the individual completes household clean energy transition, otherwise Clean_use_it_ = 0. Control_it_ denotes a series of control variables, with μ_ρ_ and λ_t_ representing province fixed effects and time fixed effects. ε_it_ represents the random error term, and the coefficient β_1_ reflects the effect of household clean energy transition on medical costs. Regression standard errors are clustered at the individual level, taking into account the correlation between sample individuals.

#### 3.3.2 Parallel trend model

The presence of a consistent trend between the treatment and control groups prior to the implementation of the policy intervention is a critical condition for ensuring the validity of the DID estimation. In this subsection, we employ the event study methodology, as proposed by (23), to evaluate the treatment effect of the policy. The specific model formulation is outlined in Equation 2. The timing of individual household energy transitions is aligned with the number of survey periods in the CFPS, designating the moment of transition as period 0. We establish three periods both preceding and following the reform. For instance, if a household completes its transition to clean energy in 2016, that year is designated as period 0 for that household. The years 2014, 2018, and 2020 are then classified as periods −1, 1, and 2, respectively, following the transition.


(2) $$Y_{\mathrm{it}}=\beta_{0}+\beta_{1}\sum_{\theta =-3}^{3}\text{Clean}\_\mathrm{us}e_{\mathrm{it}}^{\theta =t-t_{0}}+\beta_{2}\text{Contro}l_{\mathrm{it}}+\mu_{\rho }+\lambda_{t}+\varepsilon_{\mathrm{it}}$$

Where t_0_ is the period when the respondent completed the household energy transition, and *θ* is the CFPS survey period interval, Clean_use =itθ0 when θ < 0, otherwise Clean_use =itθ1 .

#### 3.3.3 Indirect placebo model

To mitigate the potential influence of other randomized factors in estimating the results of this study, we employed the methodology of an indirect placebo test (24–26), the details of which can be found in Equation 3 for the construction of the model. The estimation of Clean_use_it_ is as follows:


(3) $$\beta^{\wedge }=\beta +\sigma \frac{\mathrm{cov}(\text{Clean}\_\mathrm{us}e_{\mathrm{it}},\varepsilon_{\mathrm{it}}|S)}{\mathrm{var}(\text{Clean}\_\mathrm{us}e_{\mathrm{it}}|S)}$$

In this model, S represents a comprehensive set of non-core independent variables (or control variables), while *σ* denotes factors that have not yet been directly observed but may potentially impact residents’ medical costs. If the value of σ is 0, it indicates that these potential unobserved factors do not significantly interfere with our estimation results in any substantial way, thus verifying that the estimate of the coefficient *β* is fair and unbiased. Since it is not possible to validate σ directly, this paper employs an indirect placebo methodology. The methodology is that we randomly generating a simulated data sample of respondents who are completing household clean energy transition within a hypothetical scenario. However, due to its stochastic characteristics, the theoretical expectation for the estimate of the effect of policy implementation, denoted as β*, is that it should equal 0. Under this premise, any observed deviation of β* from 0 suggests that *σ* is also not equal to 0, indicating the presence of bias in the estimate.

#### 3.3.4 Two-step approach

To assess the mediating effect, we employ a two-step approach as outlined by Jiang (27). This method involves the formulation of Equations 4 and 5, in which both health_it_ and income_it_ act as mediating variables, representing the health effect and income effect, respectively. The presence of a significant regression coefficient β_1_ in the staggered DID model, along with a significant α_1_, indicates the existence of a mediating effect. This suggests that the transition to clean energy within households impacts individual medical costs through both health and income effects. The model is structured as follows:


(4) $$\text{healt}h_{\mathrm{it}}=\alpha_{0}+\alpha_{1}\text{Clean}\_\mathrm{ue}s_{\mathrm{it}}+\alpha_{2}\text{Contro}l_{\mathrm{it}}+\mu_{\rho }+\lambda_{t}+\varepsilon_{\mathrm{it}}$$

(5) $$\text{incom}e_{\mathrm{it}}=\alpha_{0}+\alpha_{1}\text{Clean}\_\mathrm{ue}s_{\mathrm{it}}+\alpha_{2}\text{Contro}l_{\mathrm{it}}+\mu_{\rho }+\lambda_{t}+\varepsilon_{\mathrm{it}}$$

## 4 Regression results

### 4.1 Baseline regression results

Table 3 presents baseline estimates regarding the influence of household clean energy transition on medical expenditures. Specifically, columns (1) and (2) illustrate the direct impacts of this transition on medical expenses, while column (2) additionally incorporates fixed effects associated with both province and time. The estimated coefficients in both columns are positive and statistically significant at the 1 percent level. Columns (3) and (4) introduce control variables, with column (4) further adjusting for province and year fixed effects. The findings indicate that households utilizing clean energy experience a substantial reduction of 16.1 percent in medical costs. Overall, the results in Table 3 underscore the significant impact of the household energy transition on reducing medical expenses within the population.

**Table 3:** Descriptive statistics.

Variables	(1)	(2)	(3)	(4)
	fp511	fp511	fp511	fp511
did	−0.215*** (0.046)	−0.129*** (0.049)	−0.219*** (0.046)	−0.161*** (0.048)
gender			0.003 (0.048)	−0.011 (0.047)
age			0.157*** (0.028)	0.147*** (0.028)
age2			−0.278*** (0.045)	−0.253*** (0.045)
qa301			0.027 (0.059)	0.019 (0.061)
edu			0.001 (0.005)	0.011* (0.006)
qp605			0.409*** (0.077)	0.298*** (0.076)
qp201			−0.200*** (0.021)	−0.185*** (0.021)
qp203			0.021 (0.014)	0.028** (0.014)
qp204			0.687*** (0.071)	0.639*** (0.071)
Constant	5.564*** (0.033)	5.517*** (0.035)	3.659*** (0.440)	3.633*** (0.436)
Pro	N	Y	N	Y
Year	N	Y	N	Y
Observations	15,420	15,419	15,420	15,419
R^2^	0.002	0.026	0.025	0.045

***, ** and * denote significance levels of 1 per cent, 5 per cent and 10 per cent, respectively, with robust standard errors in parentheses.

In the analysis of control variables, it was observed that individual age exhibits a non-linear U-shaped relationship with medical costs at the 1% significance level, with individuals in the middle age range incurring higher medical expenses. The variable representing education demonstrates a positive coefficient, suggesting that individuals with greater educational attainment tend to have higher medical costs. Furthermore, the variable qp605 shows a significantly positive coefficient, indicating that participation in basic medical insurance is associated with increased medical costs This may be due to the fact that insured individuals tend to utilize medical services more frequently than those who are not insured. Additionally, the coefficient for qp201 is significantly negative, revealing an inverse relationship between personal health status and medical costs. Conversely, the coefficient for qp203 is positive, indicating that individuals with chronic illnesses or poorer health conditions incur higher medical costs. Lastly, the coefficient for qp204 is also positive, suggesting that individuals opting for care at more specialized medical facilities face higher medical expenses.

### 4.2 Robustness tests

#### 4.2.1 PSM-DID estimation

Given that individuals live in diverse regions with varying economic conditions and personal resources, extraneous factors may influence the likelihood of a household’s transition to clean energy, potentially introducing bias into the regression outcomes. To address the issue of self-selection within the sample, we employ the PSM-DID methodology for regression analysis. Following a comprehensive evaluation of various matching techniques, we find that 1:1 nearest neighbor matching yields the most favorable results. The regression findings are presented in Table 4, which shows that the coefficient for PSM-DID in column (1) is significantly positive, indicating a 16.3% reduction in medical expenditures for individuals who have successfully transitioned their households to clean energy. This result affirms the robustness of the regression analysis.

**Table 4:** Robustness tests.

Variables	(1)	(2)
	PSM-DID	Burden
did	−0.163*** (0.048)	−0.033*** (0.005)
Constant	3.667*** (0.435)	0.402*** (0.045)
Controls	Y	Y
Pro	Y	Y
Year	Y	Y
Observations	15,353	15,379
R^2^	0.046	0.061

***, ** and * denote significance levels of 1 per cent, 5 per cent and 10 per cent, respectively, with robust standard errors in parentheses.

#### 4.2.2 Changing the dependent variable

To enhance the reliability of the regression findings, we introduce a new dependent variable, Burden, which represents the medical burden experienced by respondents. This variable is quantified as the ratio of medical costs to total costs. The regression outcomes are presented in column (2) of Table 4, Burden is significantly negative. This indicates that household clean energy transition substantially alleviates household medical burden, thereby reinforcing the robustness of the obtained results.

#### 4.2.3 Parallel trends test

**Figure 3:**
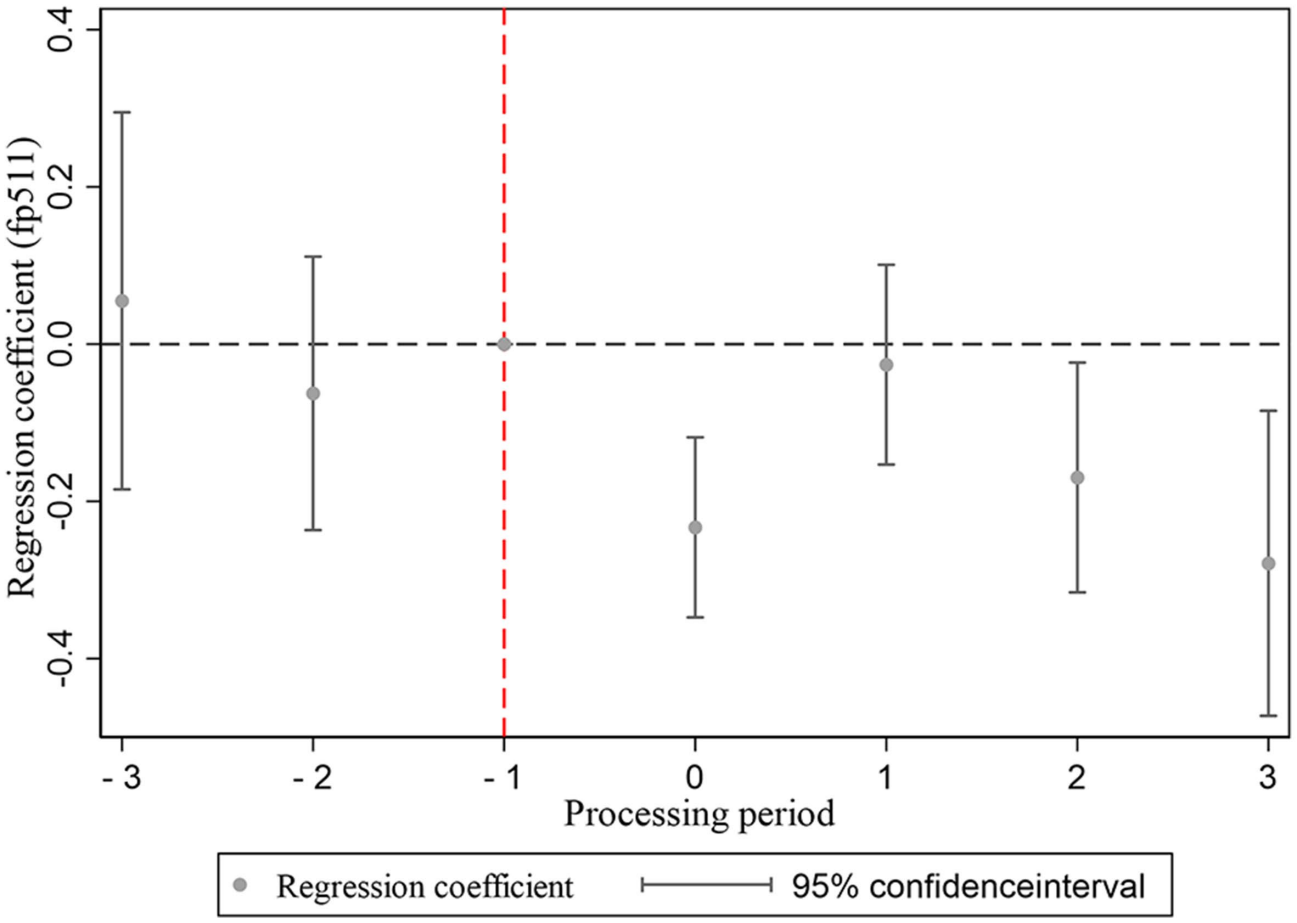
Balance trend test chart.

In order to verify the validity of the benchmark regression, this study examines the impact of dynamic changes in households’ average residential medical expenditures before and after the clean energy transition, as outlined in Equation 2. We designate the −1 period, during which individual households complete their transition to clean energy, as the baseline period. Before conducting the parallel trend test, we performed an F-test to verify whether the parallel trend assumption held before the household energy transition. The results are presented in Table 5. We found that the *p*-values of the F-tests for the period one period prior to the transition, two periods prior to the transition, and three periods prior to the transition were all greater than 0.1. Consequently, we fail to reject the null hypothesis, indicating that the trends in the treatment and control groups were parallel prior to the household energy transition. The findings are illustrated in Figure 3. Prior to the implementation of the household clean energy transition, the medical expenses incurred by residents in both the control and treatment groups demonstrated a notable degree of stability, with no significant disparities observed, suggesting a strong parallelism in the trends of medical expenditures between the two groups before the transition began. This observation supports the validity of the DID methodology employed in this research. Following the initiation of the household clean energy transition, there is a noticeable and sustained decline in residents’ medical expenses over time, indicating a significant and enduring reduction in medical costs associated with the clean energy transition. This outcome further corroborates the robustness of the regression analysis.

**Table 5:** F test.

Period	(1)	(2)
	F test	*p* value
Pre1	0.69	0.4066
Pre2	0.01	0.9211
Pre3	1.14	0.2856

***, ** and * denote significance levels of 1 per cent, 5 per cent and 10 per cent, respectively, with robust standard errors in parentheses.

#### 4.2.4 Placebo test

**Figure 4:**
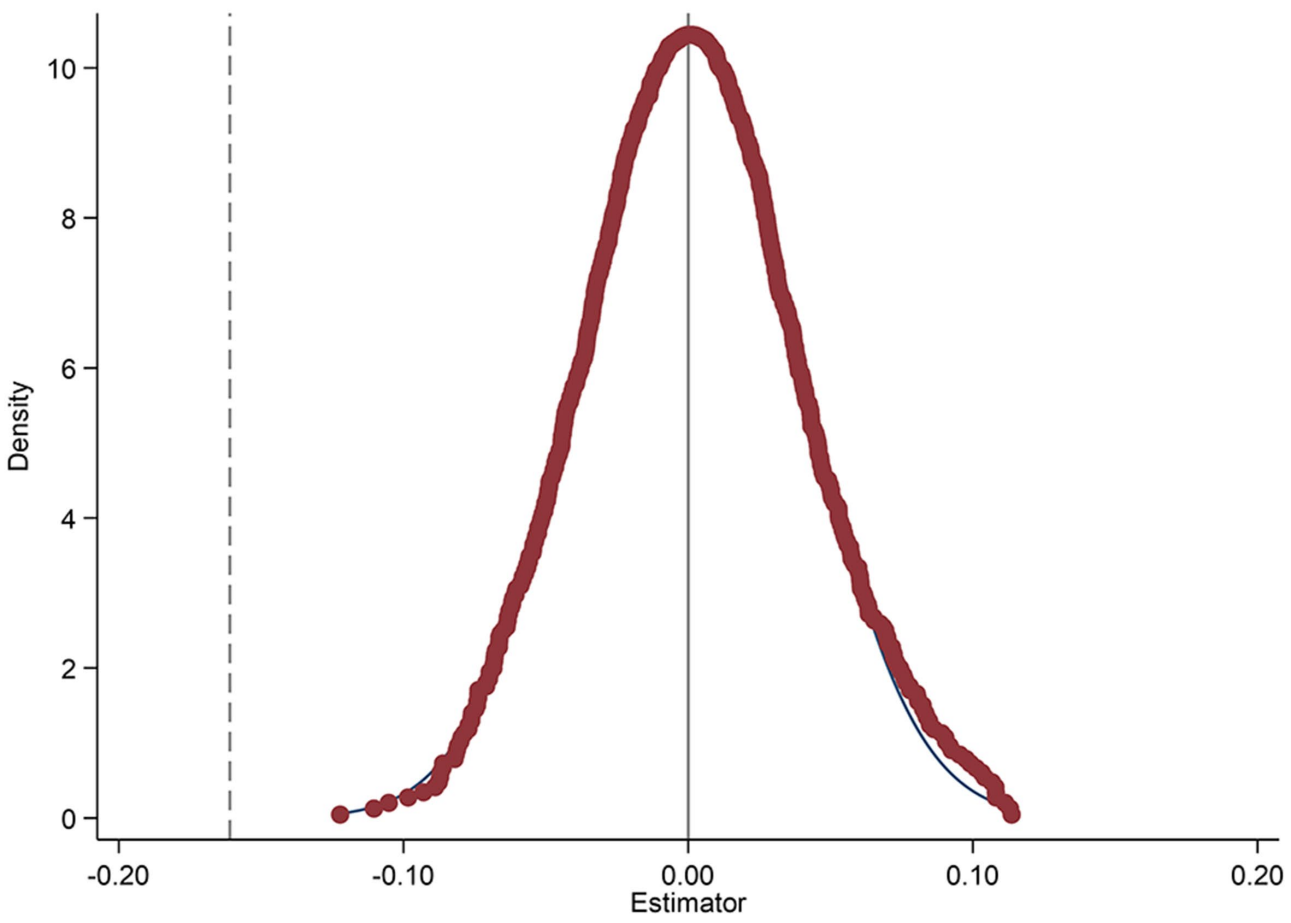
Placebo effect test figure.

Households that adopt clean energy at an earlier stage may have more favorable perceptions of their economic status and health literacy. Consequently, there may be factors beyond clean energy policies that contribute to a significant reduction in medical expenditures for individuals who have successfully transitioned their households to clean energy. In order to mitigate the influence of these factors, we employed an indirect placebo method. To increase the identification power of this placebo test, it is repeated 1,000 times. Figure 4 plots the distribution of the estimated policy effect β* from 1,000 runs. As shown in Figure 4, the values of β* demonstrate a central tendency around 0 and are normally distributed. When the estimated coefficients significantly deviate from this normal range and display clear outliers, it further reinforces the robustness and reliability of the estimation results presented in this paper. Typically, if the estimation process is not influenced by unusual factors or biases, the estimated coefficients should cluster around the true value (0 in this case) without extreme outliers. Therefore, the presence of these outliers indicates, from another viewpoint, that our estimation method effectively captures the true relationship between the variables and remains robust despite potential confounding factors.

### 4.3 Mechanism analysis

In this section, we examine the existence of health and income effects associated with the reduction of medical costs resulting from household clean energy transition. Specifically, we utilize changes in health status as a metric for the health effect, denoted as “health.” In accordance with the CFPS inquiry regarding changes in health status over the past year, we assigned a value of 1 to the variable representing health for respondents who indicated that their health “has gotten better,” while assigning a value of 0 to those who reported that their health “has not changed” or “has gotten worse.” To estimate the health effect, we utilized a Logit model. Regarding the income effect, our analysis centers on the logarithmic transformation of per capita household income, referred to as “income,” and estimate the effect using Two-way fixed effects model.

The regression results pertaining to the mediating effect are presented in Table 6. The coefficient for health_it_ presented in column (1) indicates a statistically significant positive relationship, suggesting that the transition of households to clean energy substantially increases the likelihood of health improvements among individuals, with a noted increase of 19.7% in this probability. This result aligns with the empirical results reported by Wu et al. (3), thereby reinforcing the foundation of our research. It not only supports the validity of Hypothesis 1, which posits that the adoption and promotion of clean energy within households can effectively reduce medical expenditures through the mechanism of enhancing physical health, but it also establishes a coherent logical framework: transition to clean energy → health improvement → reduction in medical costs.

**Table 6:** Mediating effect test.

Variables	(1)	(2)
	Health	Income
did	0.197*** (0.065)	0.290*** (0.019)
Constant	1.261** (0.547)	8.309*** (0.180)
Controls	Y	Y
Pro	Y	Y
Year	Y	Y
Observations	15,401	14,671
Pseudo R^2^	0.0458	
R^2^		0.352

***, ** and * denote significance levels of 1 per cent, 5 per cent and 10 per cent, respectively, with robust standard errors in parentheses.

Furthermore, the coefficient for income_it_ presented in column (2) demonstrates a statistically significant positive relationship, indicating that a household’s transition to clean energy has a favorable effect on an individual’s income status. Specifically, the likelihood of an improvement in an individual’s income status increases by 29 percent following the household’s adoption of clean energy practices. This finding corroborates hypothesis 2, which asserts that household clean energy transition can contribute to reduced medical expenses by enhancing individuals’ income levels. This positive correlation not only underscores the potential economic advantages associated with the clean energy transition but also provides new insights into its implications for social welfare and individual economic circumstances.

### 4.4 Heterogeneity analysis

Given the regional disparities in individual lifestyles, variations in human capital, differences in homeownership, and distinctions in household status, it is probable that these factors influence the fuel choices of households. This section therefore conducts a more in-depth analysis of how household transitions to clean energy relate to regional heterogeneity, educational disparities, homeownership variations, and differences in household size, particularly in the context of reducing residential medical expenditures.

#### 4.4.1 Regional heterogeneity

Narasimha Rao and Reddy (28) investigated the influence of household locational characteristics on fuel selection, revealing that urban populations are more inclined to choose cleaner energy sources compared to their rural counterparts. This discrepancy can be ascribed to variations in fuel accessibility and availability. Accordingly, we categorize the sample into two distinct groups based on household location characteristics—rural and urban areas—to analyze the effects of the clean energy transition on medical costs in each context separately. The results of the regression analysis, detailed in Table 7, indicate that the clean energy transition has a more pronounced effect on reducing medical costs for the rural population than for the urban population. A plausible explanation for this finding is that rural areas often face economic disadvantages, leading to poorer health outcomes among residents, who may encounter greater health risks and illnesses compared to those in urban settings (29). Thus, the adverse effects of the transition to clean energy on medical costs are more significant for rural residents.

**Table 7:** Regional heterogeneity.

Variables	(1)	(2)
	Rural areas	Urban area
did	−0.174*** (0.055)	−0.145 (0.103)
Constant	3.530*** (0.491)	4.283*** (0.900)
Controls	Y	Y
Pro	Y	Y
Year	Y	Y
Observations	Y	Y
R^2^	0.047	0.058

***, ** and * denote significance levels of 1 per cent, 5 per cent and 10 per cent, respectively, with robust standard errors in parentheses.

#### 4.4.2 Educational heterogeneity

In the analysis of the determinants influencing household energy selections, Rahut et al. (30) and Paudel et al. (31) observed that households with higher levels of education exhibit a greater propensity to choose cleaner energy alternatives. Building on their research, we propose that the effects of household transitions to clean energy on medical expenditures may vary according to individuals’ educational levels. Drawing on insights from prior studies, we categorized the sample into two groups based on educational attainment, using the 9-year compulsory education threshold as the cutoff criterion (32, 33). The results of the educational heterogeneity analysis are presented in Table 8. The coefficient in column (1) is significantly positive, indicating that the transition to clean energy within households resulted in a 21.2% reduction in medical costs for the low-education group; however, this effect does not reach statistical significance within the high-education group. This discrepancy may be attributed to the high human capital and favorable economic and health conditions of the high-education group (34, 35), which diminishes the significance of the impact of household clean energy transitions on their medical expenditure.

**Table 8:** Educational heterogeneity.

Variables	(1)	(2)
	Low education	High education
did	−0.212*** (0.063)	−0.109 (0.071)
Constant	3.862*** (0.506)	3.054* (0.790)
Controls	Y	Y
Pro	Y	Y
Year	Y	Y
Observations	8,127	7,101
R^2^	0.056	0.043

***, ** and * denote significance levels of 1 per cent, 5 per cent and 10 per cent, respectively, with robust standard errors in parentheses.

#### 4.4.3 Homeownership heterogeneity

The investment decisions of individuals regarding modern fuels are significantly influenced by lease agreements and the condition of their residences (36). Homeowners, who possess full or partial property rights, are generally more motivated to implement improvements to their living spaces. In contrast, individuals in leasehold arrangements, who occupy temporary housing, tend to be hesitant to allocate time and financial resources toward maintenance efforts. Therefore, this study categorizes the sample into two distinct groups based on homeownership status: homeowners and non-homeowners. The results of the homeownership heterogeneity analysis, presented in Table 9, indicate that the transition to home energy systems led to a 13.5 percent decrease in medical costs for homeowners, while no significant impact was observed for non-homeowners.

**Table 9:** Homeownership heterogeneity.

Variables	(1)	(2)
	Having house property rights	NO house property rights
did	−0.135*** (0.051)	−0.081 (0.136)
Constant	4.001*** (0.449)	−0.785 (1.485)
Controls	Y	Y
Pro	Y	Y
Year	Y	Y
Observations	13,085	2,318
R^2^	0.047	0.065

***, ** and * denote significance levels of 1 per cent, 5 per cent and 10 per cent, respectively, with robust standard errors in parentheses.

#### 4.4.4 Family size heterogeneity

Household size is a critical determinant of fuel selection in domestic settings (37, 38). An increase in the number of individuals residing in a household typically necessitates a greater quantity of food preparation, which can subsequently elevate household fuel expenditures. Generally, cleaner fuel options tend to be more expensive than solid fuels; thus, households with varying demographic characteristics may choose different fuel types based on their income levels. This variability in fuel choice can significantly influence the likelihood of a household undergoing an energy transition. Accordingly, this study categorizes the entire sample into three distinct groups based on household size and examines the implications of household energy transitions on medical expenditures across these groups. The results of the family size heterogeneity analysis are presented in Table 10. The coefficient in column (2) reveals a statistically significant negative relationship, while the coefficients in the other columns do not demonstrate significance. This suggests that the clean energy transition has a notably adverse effect on medical costs for households comprising 3 to 5 members. In contrast, for households with 1 to 2 members compared to those with more than 5 members, the higher expenses associated with cleaner fuels may lead to a crowding-out effect on medical costs, resulting in non-significant regression outcomes.

**Table 10:** Family size heterogeneity.

Variables	(1)	(2)	(3)
	1–2 members	3–5 members	Above 5 members
did	−0.186 (0.242)	−0.100* (0.061)	−0.052 (0.073)
Constant	−1.136 (2.669)	3.336*** (0.536)	5.584*** (0.676)
Controls	Y	Y	Y
Pro	Y	Y	Y
Year	Y	Y	Y
Observations	1,308	9,712	4,395
R^2^	0.065	0.054	0.049

***, ** and * denote significance levels of 1 per cent, 5 per cent and 10 per cent, respectively, with robust standard errors in parentheses.

## 5 Conclusions and policy implications

Using the implementation of the APCP as a quasi-natural experiment, this paper investigates the effects of household clean energy transition on medical expenditures with a staggered DID design and micro survey data. The findings indicate that household clean energy transition significantly reduces medical costs, with a notable decrease of 16.1% observed among individuals who have completed this transition. This result survives various robustness checks. Mechanism analyses reveal that the reduction in medical costs associated with the clean energy transition is primarily mediated by improvements in health outcomes and increases in income. Furthermore, heterogeneity analyses indicate that the reduction in medical costs is especially significant among rural residents, individuals with lower educational attainment, homeowners, and households comprising three to five members. Based on these findings, the study proposes several policy implications.

First, to effectively facilitate household transitions to clean energy, it is essential to enhance public awareness of clean energy and its beneficial effects on health and income. By fostering a greater understanding of these advantages, individuals may be more inclined to voluntarily engage in clean energy adoption.

Second, governments should enhance clean energy subsidies for rural residents and individuals with lower levels of education to boost their purchasing power and promote the transition to household clean energy.

Last, policy formulation must take into account the costs associated with clean energy and the energy consumption patterns of various household structures, ensuring that these measures effectively support the most disadvantaged groups and thereby improve energy consumption patterns while enhancing the overall quality of life for the population.

## Data Availability

Publicly available datasets were analyzed in this study. This data can be found at: Publicly available datasets were analyzed in this study. This data can be found at: http://www.isss.pku.edu.cn/cfps/index.htm.
