# Supplementary material for: Can household clean energy transition reduce medical expenditures? Evidence from China
Source: Front Public Health. 2025 Aug 15;13:1524444. doi: 10.3389/fpubh.2025.1524444 (PMC12394507; doi:10.3389/fpubh.2025.1524444)
Supplement: Supplementary file 1 [file Data_Sheet_1.docx]

***Supplementary Material***


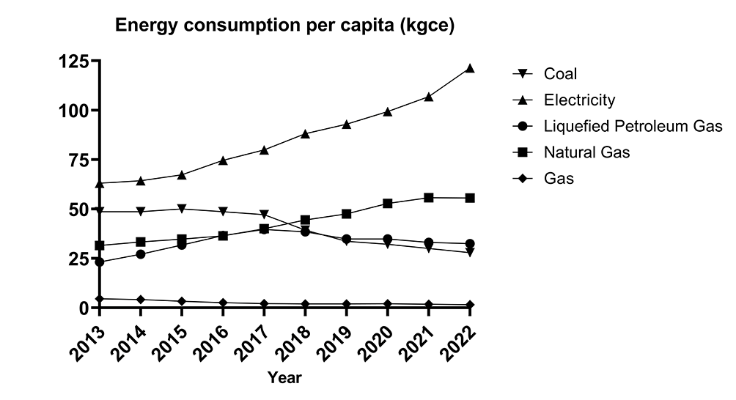


**Supplementary Figure 1.** Per capita domestic energy consumption, China Energy Statistics Yearbook, 2013-2023


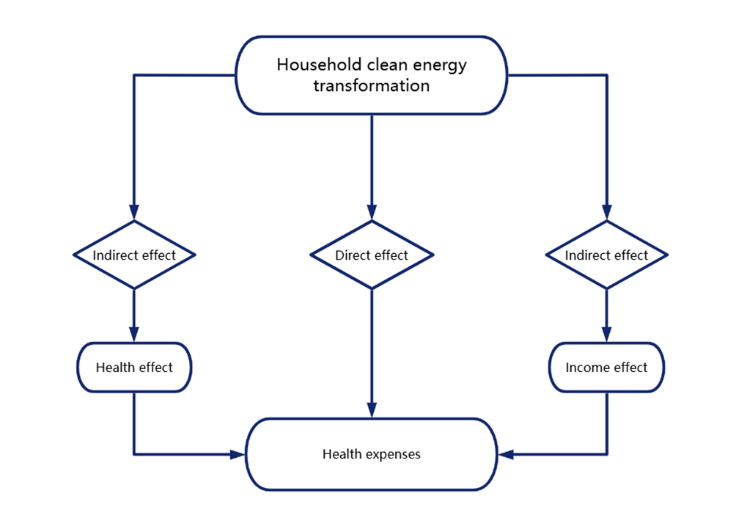


**Supplementary Figure 2.** Mediating effect


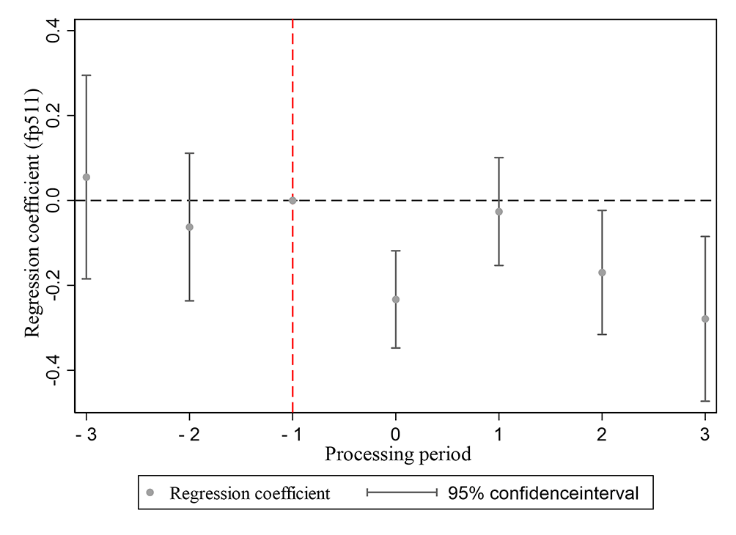


**Supplementary Figure 3.** Parallel trends test


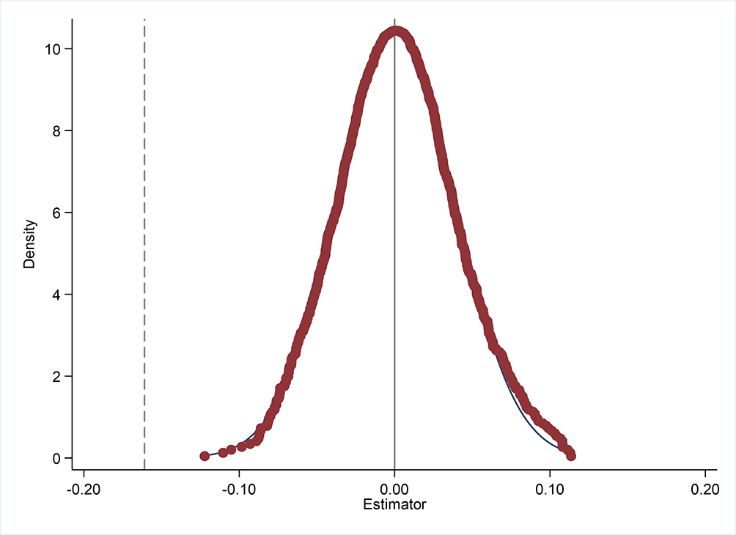


**Supplementary Figure 4.** Placebo test
